# Genome-wide scans for detecting the selection signature of the Jeju-island native pig in Korea

**DOI:** 10.5713/ajas.19.0026

**Published:** 2019-07-01

**Authors:** Young-Sup Lee, Donghyun Shin, Kyeong-Hye Won, Dae Cheol Kim, Sang Chul Lee, Ki-Duk Song

**Affiliations:** 1 Department of Animal Biotechnology, Jeonbuk National University, Jeonju 54896, Korea; 2 The Animal Molecular Genetics and Breeding Center, Jeonbuk National University, Jeonju 54896, Korea; 3 Livestock Promotion Agency, Jeju Special Self-Governing Province, Jeju 63122, Korea; 4 Cronex Co., Cheongju 28174, Korea

**Keywords:** Jeju Island Native Pig, Berkshire Pig, Linkage Disequilibrium, Population Differentiation Statistic, Selection Signatures, Yorkshire Pig

## Abstract

**Objective:**

The Jeju native pig (JNP) found on the Jeju Island of Korea is a unique black pig known for high-quality meat. To investigate the genetic uniqueness of JNP, we analyzed the selection signature of the JNP in comparison to commercial pigs such as Berkshire and Yorkshire pigs.

**Methods:**

We surveyed the genetic diversity to identify the genetic stability of the JNP, using the linkage disequilibrium method. A selective sweep of the JNP was performed to identify the selection signatures. To do so, the population differentiation measure, Weir-Cockerham’s F_st_ was utilized. This statistic directly measures the population differentiation at the variant level. Additionally, we investigated the gene ontologies (GOs) and genetic features.

**Results:**

Compared to the Berkshire and Yorkshire pigs, the JNP had lower genetic diversity in terms of linkage disequilibrium decays. We summarized the selection signatures of the JNP as GO. In the JNP and Berkshire pigs, the most enriched GO terms were epithelium development and neuron-related. Considering the JNP and Yorkshire pigs, cellular response to oxygen-containing compound and generation of neurons were the most enriched GO.

**Conclusion:**

The selection signatures of the JNP were identified through the population differentiation statistic. The genes with possible selection signatures are expected to play a role in JNP’s unique pork quality.

## INTRODUCTION

Of the various pig breeds, the Jeju native pig (JNP) can be considered as one of the representatives of Korean native black pig (KNBP) [1]. The JNP has black skin, erect, unfolded ears and is well-known for its tender and juicy meat. Consumers prefer JNP meat because of its superior taste, tenderness and marbling quality compared to the meat from western breeds [2]. JNP meat can be regarded as one of the best pork in Korea. The uniqueness of the JNP meat may come from the distinct climate of Jeju Island of Korea and the life pattern of the people of Jeju Island. Notably, the winter temperature on Jeju Island remains above zero and the JNP are used to dispose of human waste. These factors might change the allele patterns in the JNP genome compared to other pig breeds. JNP has problems of lower feed efficiency and smaller litter sizes as compared to Berkshire pig breeds [2]. Thus to understand the uniqueness of the JNP and JNP pork quality, a comparative genomic study between the JNP and other commercial pig breeds like Berkshire or Yorkshire pigs, is needed. In a previous study, Ghosh et al [3] used RNA-seq to identify pork quality-related genes.

Population differentiation (subdivision) is a fundamental process of evolution and its infer ence is required for genetics, phylogeography and conservation biology. It can be recognized as “fundamental” because every species unavoidably undergoes population differentiation, and it may lead to new speciation or extinction under certain conditions. Additionally, genetic differentiation of populations can be the result of uneven (nonrandom) spatial distribution of genetic variation and allele frequencies in a species. The fixation index, F_st_, is the measure of population differentiation [4].

In previous study, Kim et al [5], used the cross-population extended haplotype homozygosity (XP-EHH) and cross population composite likelihood ratio (XP-CLR) to identify the putative selection signature causes of meat quality of JNP. The XP-EHH statistic examines the haplotype difference between two populations and detects alleles that have increased in frequency at the point of fixation or near-fixation. The haplotypes that are frequent and have longer than expected values are regarded as being positively selected. Alternatively, XP-CLR evaluates the allele frequency differentiation between two populations to assess the candidate regions of the selective sweeps. Selective sweep regions can be considered to be highly positive-selected. Oh et al [6], used the F_st_ of the microsatellite markers of JNP to assess meat quality. Our approach was to use Fst of single nucleotide polymorphism (SNP) markers.

In this study, we aimed to reveal genomic differences and the selective sweep regions of the JNP in comparison to the Berkshire and Yorkshire breeds using the fixation index. The selection signatures can be identified through the selective sweep analysis of the genomic regions like population differentiation. The selection signature of the JNP was revealed through the population differentiation statistic.

## MATERIALS AND METHODS

### Ethical statement

The experimental procedure followed Institutional Animal Care and Use Committee regulations (CRONEX-IACUC 201810005).

### Data preparation

We randomly sampled 50 JNP from the Jeju Livestock Promotion Institute (Jeju, Korea), 151 Yorkshire pigs from GGP farms, and 67 Berkshire pigs from Dasan Breeding Farm (Namwon, Korea). The genomic DNAs of the individuals were genotyped using an Illumina Porcine 60 K SNP Beadchip (Illumina, San Diego, CA, USA) following the standard protocol. We merged the three pig breeds’ data using vcftools (vcftools.sourceforge.net) and obtained 62,551 SNPs [7]. For the quality control, we excluded the SNPs with minor allele frequency (<0.05) and Hardy-Weinberg equilibrium (p<0.0001) and genotyping call rate (<0.05).

### Structure analysis and linkage disequilibrium

To understand the characteristics of the JNP, Berkshire and Yorkshire pig populations, structure analysis was performed with the structure program [8]. We set the 5,000 iterations after the burn-in 5,000 iterations and K = 3, 4, and 5. We evaluated the JNP’s purity and structure analysis showed that the JNP was mixed with Berkshire in part. Thus, we eliminated impure JNP individuals for the next analysis.

The correlation coefficient of the linkage disequilibrium (r^2^) was computed to assess the genetic diversity of the JNP, Berkshire and Yorkshire pigs. Linkage disequilibrium is the nonrandom associations of alleles at two loci, A and B. Linkage disequilibrium is usually used to assess the genetic diversity of a given population, estimation of effective population size and population genetic stability. Using the R package “LDcorSV”, we examined all pairwise r^2^ decays with 100 SNP bins along the physical distance [9]. The formula based on this is as follows:

(1) $$r^{2}=\frac{D_{AB}^{2}}{P_{A}P_{a}P_{B}P_{b}}$$

where $D_{AB}^{2}$ is the linkage disequilibrium of two alleles A, B and *P_X_* is the frequency of major allele X and *P_X_* is the frequency of minor allele x.

### Population differentiation analysis

We used the population differentiation statistic (F_st_) to find selection signatures of the JNP breeds. The reference pig breeds were Berkshire and Yorkshire. Berkshire and Yorkshire pigs are worldwide commercial pig breeds that we felt provided an adequate comparison to the JNPs. Among various population differentiation statistics, the statistic was the Weir-Cockerham F_st_ in vcftools [10–12]. The Weir-Cockerham F_st_ formula is as follows:

(2) $$F_{ST}=\frac{s^{2}}{\bar{p}(1-\bar{p})}$$

Where *s*^2^ is the sample variance of the allele frequency of A over subpopulations and *p̄* is the average of the allele frequency of A in the total populations of the biallelic system [11,13].

## RESULTS

### Genotype data

Among the 62,551 autosomal SNPs genotyped in this analysis, 46,505 in JNP-Berkshire and 44,306 in JNP-Yorkshire remained after quality control. After filtering and imputation, the number of SNPs per autosome ranged from 1,085 to 6,000 in the JNP-Berkshire data and from 1,030 to 5,864 in the JNP-Yorkshire data, and this value was closely related to the chromosome length and total number of SNPs, as shown in Supplementary Figure S1. The remaining minor allele frequency of SNPs exhibited a uniform distribution, with an average of 0.42±0.22 (standard deviation [SD]). The mean distance between adjacent SNP pairs from this analysis was 50,134±206,871 (SD).

### Linkage disequilibrium of Jeju native pig

**Figure 1:**
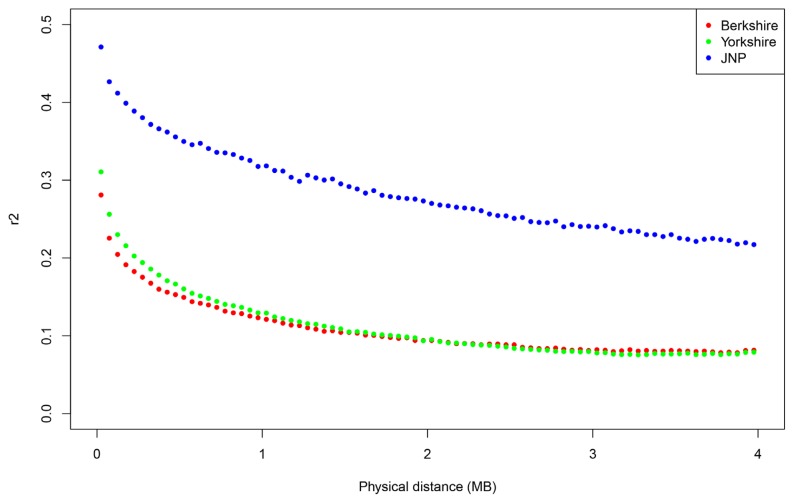
The correlation coefficient of linkage disequilibrium (r^2^) decay plotted against physical distance. The Jeju native pig (JNP) decayed much smoother than the Berkshire and Yorkshire pigs, reflecting the genetic diversity of the JNP is lower than that of the Berkshire and Yorkshire pig populations.

**Figure 2:**
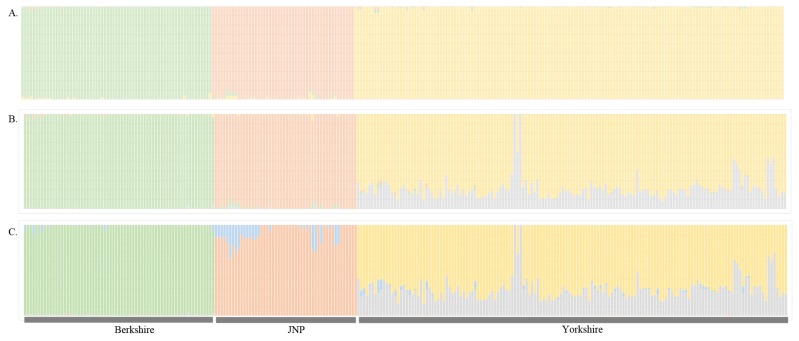
Structure analysis to evaluate the purity of the population (A, K = 3; B, K = 4; C, K = 5).

We evaluated the structure distinctness of the JNP, Berkshire and Yorkshire pigs, and the linkage disequilibrium decays were examined to assess the genetic diversity. We used the R package “LDcorSV” to examine the linkage disequilibrium. All pairwise linkage disequilibriums were calculated within adjacent 100 SNPs. Figure 1 shows *r*^2^ decay with the physical distance in the JNP, Yorkshire and Berkshire pigs. According to Figure 1, the genetic diversity of the JNP were considerably lower than that of the Yorkshire and Berkshire pigs. Additionally, this lower genetic diversity reflects that the number of JNPs has decreased since South Korea has been modernized after the 20th century. We analyzed the population differentiation to examine the population structure of the JNP, Berkshire and Yorkshire pig breeds. Figure 2 shows the result of structure analysis after pre-structure analysis was used to identify and eliminate the impurity of the JNP and other pigs using the case K = 3.

### Population differentiation analysis

**Figure 3:**
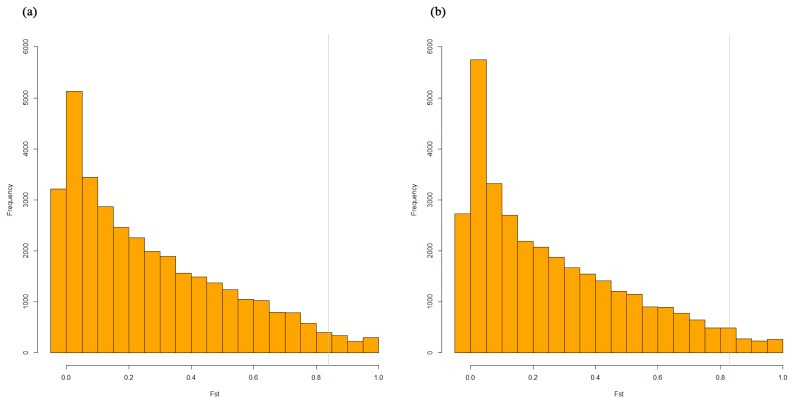
The histogram of the population differentiation statistic in Jeju native pig (JNP) vs Berkshire pigs and JNP vs Yorkshire pigs. (a) JNP vs Berkshire and (b) JNP vs Yorkshire. The vertical gray line shows that the right part was used for gene ontology (GO) analysis.

To detect and characterize of the JNP’s selection signatures, we used the population differentiation statistic, F_st_ in which the selection signature between two compared breeds can be revealed. F_st_ is the widely used population differentiation statistic in selective sweep analysis. Practically, we used the Weir-Cockerham’s F_st_ statistic. The mean and standard deviation in F_st_ analysis of JNP vs Berkshire and JNP vs Yorkshire were 0.27±0.25 and 0.26±0.25, respectively. Figure 3 shows the distribution of F_st_ of the JNP, Berkshire and Yorkshire pigs. The frequencies of the F_st_ values decreased with increasing F_st_ values, and the gray line represents the cutoff F_st_ (top 5% genes) for gene ontology (GO) analysis. Supplementary file 1 consists of the significant gene’s SNP list and its allele frequencies. Additionally, this file shows the discrepancy of the allele frequency of JNP, Berkshire and JNP, Yorkshire pigs.

### Genes with high F_st_ values

The high F_st_ value (F_st_ =1) genes in the JNP vs Berkshire were tight junction protein 1 (*TJP1*), 4-aminobutyrate aminotransferase (*ABAT*), matrix metallopeptidase 25 (*MMP25*), methionine sulfoxide reductase B1 (*MSRB1*), osteoglycin (*OGN*), plexin C1, and ubiquitin protein ligase E3B (*UBE3B*). The high F_st_ value genes in the JNP vs Yorkshire were interferon induced protein 44 and its paralog (*IFI44* and *IFI44L*), DEAD-box helicase 59 (*DDX59*), cathepsin O, and prostaglandin E receptor 3 (*PTGER3*).

### Functional classification and biological pathway analysis

We performed gene matching with the high F_st_ SNPs. After gene matching of SNPs using ensemble gene catalogue (www.ensembl.org), the top 5% of genes were used in DAVID GO analysis (The Database for Annotation, Visualization and Integrated Discovery v6.8; https://david.ncifcrf.gov/). The DAVID analysis includes three types of classification (biological process, molecular function, and cellular component). Practically, we used the biological process to query the selective sweep genes’ GO. For the selective sweep genes’ GO, the multiple correction and p-value approach (cutoff p-value 0.01 or 0.05) was not adequate in our analysis because it was very harsh. Table 1 and 2 show the gene ontologies (GO) of the JNP vs Berkshire and JNP vs Yorkshire pigs, respectively. For the JNP vs Berkshire pigs, epithelium development and neurogenesis were enriched and for the JNP vs Yorkshire pigs, cellular response to oxygen-containing compound and neurogenesis were the most enriched GOs.

## DISCUSSION

### Identification of positive selected regions of Jeju native pig genomes using single nucleotide polymorphisms

There are various ways to find selection signatures in two populations such as XP-EHH, XP-CLR, F_st_, etc. These methods can be exploited by comparing two populations. XP-EHH and XP-CLR scan the genomic regions and find the selective sweep regions. XP-EHH uses the haplotype homozygosity and XP-CLR uses the population differentiation between the two object and reference population [14,15]. These methods have the advantage not computing the scores of the variants of selective sweep regions but rather comprehensively scanning the genome-wide regions. However, in the current cost restriction, sample size is a primary problem. Therefore, we instead used the F_st_ statistic which could identify the population differentiation at the direct variant level. In this study, we used SNPs data from a porcine 60K HD chip.

### Gene ontology and pathway analysis of the genes in the selective sweeps of the Jeju native pig

The significant genes in the selective sweep analysis differed considerably from Kim et al [5]. Kim et al [5] used whole genome sequencing data, whereas the SNP chip data were used in our analysis. Due to the long distance between SNPs in our dataset, we utilized another statistic, F_st_ which assesses the population differentiation at the right position of the SNPs and has the advantage to being able to find the selection signature of the SNPs based on the fixation index between two populations.

The major terms in the GO analysis were “GO:0048729~ tissue morphogenesis”, “GO:0060429~epithelium development”, and “GO:0048589~developmental growth” in the JNP vs Berkshire and “GO:1901701~cellular response to oxygen-containing compound” and “GO:0071396~cellular response to lipid”, and “GO:0022008~neurogenesis” in the JNP vs Yorkshire. The overlapped genes in JNP with Berkshire and Yorkshire pigs were 57. These kinds of genes seem to indicate the JNP’s specific genes with selective sweeps. It is of interest that some genes of the “GO:0022008~neurogenesis”, i.e., zinc finger protein 536 (*ZNF536*), FRY like transcription coactivator (*FRYL*), protein tyrosine kinase 7 (*PTK7*), protein kinase CGMP-dependent 1 (*PRKG1*), patched 1 (*PTCH1*), intraflagellar transport 140 (*IFT140*), was overlapped in JNP vs Berkshire and JNP vs Yorkshire cases. ZNF536 protein is a highly conserved zinc finger protein and is most abundant in the brain, where it negatively regulates neuronal differentiation. The FRYL plays a key role in maintaining the integrity of polarized cell extensions during morphogenesis. The *PTK7* gene encodes the proteins of a member of the receptor tyrosine kinase family and is involved in the Wnt signaling pathway. It plays a role in multiple cellular processes including polarity and adhesion. *PRKG1* isoforms play as key mediators of the nitric oxide/cGMP signaling pathway. *PTCH1* encodes a member of the patched family of proteins and is a component of the hedgehog signaling pathway. Hedgehog signaling is important in embryonic development and tumorigenesis. The encoded protein is the receptor for the secreted hedgehog ligands, which include sonic hedgehog, Indian hedgehog and desert hedgehog. Following binding by one of the hedgehog ligands, the encoded protein is trafficked away from the primary cilium, relieving inhibition of the smoothed G-protein-coupled receptor, which results in activation of downstream signaling. Mutations of this gene have been associated with basal cell nevus syndrome and holoprosencephaly. *IFT140* gene encodes one of the subunits of the IFT complex and intraflagellar transport is involved in genesis, resorption and signaling of primary cilia.

The JNP exhibits slower growth, the fat is thicker than other pigs and the litters are smaller. According to the Kyoto encyclopedia of genes and genomes (KEGG), mechanistic target of rapamycin signaling pathway was reported to regulate adiposity [16]. Additionally, the regulation of lipolysis in adipocytes is related to adipose accumulation. Wnt family member 7B (WNT7B; GO:1901701~cellular response to oxygen-containing compound in JNP vs Yorkshire pigs, ssc05205: proteoglycans in cancer in JNP vs Yorkshire pigs) is required for epithelial progenitor growth and is involved in the pancreatic development [17]. Estrogen receptor 1 (ESR1; GO:1901701~ cellular response to oxygen-containing compound in JNP vs Yorkshire pigs, ssc04917: prolactin signaling in KEGG pathway of JNP vs Berkshire pigs) is reported to be associated with litter size in the Chinese-European pig line [18]. Transforming growth factor beta-2 (TGF-beta2: GO:0022008~ neurogenesis in JNP vs Berkshire pigs) enhances connective tissue formation and wound strength in the guinea pig [19]. AKT serine/threonine kinase 3 (AKT3; ubiquitous in KEGG pathway of JNP vs Berkshire pigs) is related to reduced proliferation and facilitated differentiation of myoblasts in skeletal muscle development, whereas phosphatidylinositol-4,5-bisphosphate 3-kinase catalytic subunit alpha (PIK3CA) induces multipotency [20] (Tables 3, 4).

### High F_st_ genes’ description

TJP1 encodes a member of the membrane-associated guanylate kinase family of proteins, and it acts as a tight junction adaptor protein. Tight junctions play a role in regulating the movement of ions and macromolecules between endothelial and epithelial cells. ABAT is responsible for catabolism of gamma-aminobutyric acid, an inhibitory neurotransmitter in the central nervous system. MMP25 is involved in the breakdown of the extracellular matrix in normal physiological processes, such as embryonic development, tissue remodeling, and reproduction as well as in disease processes, such as arthritis and metastasis. MSRB1 is a member of the MSRB family functions to repair enzymes that protect proteins from oxidative stress by catalyzing the reduction of methionine-R-sulfoxides to methionines. This protein is highly expressed in the liver and kidney and it also has the highest methionine-R-sulfoxide reductase activity. OGN encodes protein that induces ectopic bone formation and may regulate osteoblast differentiation. High expression of the protein may be associated with elevated heart left ventricular mass. Plexins are transmembrane receptors for semaphorins, a large family of proteins that regulate axon guidance, cell motility and migration, and the immune response. Modification of protein with ubiquitin is an important cellular mechanism for targeting abnormal or short-lived proteins for degradation. UBE3B encoded protein are involved in ubiquitination. IFI44 and IFI44L are the interferon induced protein 44 and its paralog. Diseases associated with DDX59 include orofaciodigital syndrome V and orofaciodigital syndrome. The GO annotations related to this gene include nucleic acid binding and helicase activity. Cathepsin O enzyme is involved in cellular protein degradation and turnover. PTGER3 protein is a member of the G-protein coupled receptor family. This protein is one of four receptors identified for prostaglandin E2 and this receptor may have many biological functions, which involve digestion, the nervous system, uterine contraction activities and kidney reabsorption (www.genecards.org).

### The comparison of previous study

Kim et al [5] used XP-EHH and XP-CLR to identify the selection signatures of KNBP including JNP. There were overlapped genes such as hydroxysteroid 17-beta dehydrogense 12 (*HSD17B12*), CUB and sushi multiple domains 3, BTB domain containing 11 (*BTBD11*), ATP binding cassette subfafmily B member 11, transforming growth factor beta 2 (*TGFB2*), otoancorin, solute carrier family 4 member 10, thyroid hormone receptor beta, and *WNT7B* between our study and Kim et al’s study. *HSD17B12* gene product converts into estradiol in ovarian tissue and is involved in fatty acid elongation. JNP’s unique meat quality can be implicated in the fatty acid. Specifically, *BTBD11* was identified in every four cases of JNP vs Berkshire (with XP-EHH and XP-CLR of Kim et al [5]) and JNP vs Yorkshire (with XP-EHH and XP-CLR of Kim et al [5]).

## CONCLUSION

First, we examined the JNP’s genetic diversity using linkage disequilibrium decays. The JNP’s genetic diversity was lower than that of Berkshire and Yorkshire pigs. Second, we compared the selection signatures of JNPs to those of Berkshire and Yorkshire pigs and performed GO analysis. Some genes such as *ESR1*, *TGFB2*, and *AKT3* were related to growth, litter size and reproduction. We expect that these genes and GO genes contribute to the quality of JNP’s pork.

## Supplementary Data



## Figures and Tables

**Table 1:** The gene ontology of the selective sweep regions’ genes using F_st_ in the Jeju native pigs vs Berkshire pigs

Term	Count	p-value	Genes	Fold enrichment
GO:0048729~tissue morphogenesis	14	8.5E-05	*DVL2, PTK7, AHI1, LIN7C, PCDH15, LGR4, TGFB2, WNT2, DSP, PTCH1, KDM5B, EXT2, KDM6B, DLG1*	3.7
GO:0002009~morphogenesis of an epithelium	11	8.5E-04	*DVL2, WNT2, PTK7, AHI1, LIN7C, PCDH15, PTCH1, KDM5B, LGR4, DLG1, TGFB2*	3.6
GO:0060429~epithelium development	16	1.3E-03	*DVL2, RAD51B, PTK7, AHI1, LIN7C, PCDH15, LGR4, TGFB2, WNT2, ARG2, DSP, PTCH1, KDM5B, KDM6B, IFT140, DLG1*	2.5
GO:0048589~developmental growth	11	2.8E-03	*WNT2, FLRT1, RAD51B, PLXNC1, PTK7, PIK3CA, TXK, PCDH15, PTCH1, KDM5B, TGFB2*	3.1
GO:0022008~neurogenesis	18	3.1E-03	*ZNF536, FLRT1, PLXNC1, CCDC88A, FRYL, ZSWIM6, PTK7, AHI1, RPE65, PCDH15, PRKG1, SIRT2, TGFB2, WNT2, NDRG1, PTCH1, IFT140, LRFN2*	2.2
GO:0030182~neuron differentiation	16	3.2E-03	*ZNF536, PLXNC1, FLRT1, CCDC88A, FRYL, ZSWIM6, PTK7, AHI1, RPE65, PCDH15, PRKG1, TGFB2, WNT2, PTCH1, IFT140, LRFN2*	2.3
GO:0045216~cell-cell junction organization	6	3.5E-03	*PTPRK, OCLN, PKP4, DSP, DLG1, TGFB2*	5.8
GO:0061024~membrane organization	13	3.6E-03	*MTSS1, RAB3GAP2, OPA1, CCDC88A, PPARG, VTI1B, LIN7C, ZNF205, TGFB2, GPHN, NDRG1, PTCH1, DLG1*	2.6
GO:0044802~single-organism membrane organization	12	4.4E-03	*RAB3GAP2, MTSS1, GPHN, OPA1, PPARG, LIN7C, VTI1B, PTCH1, NDRG1, ZNF205, DLG1, TGFB2*	2.7
GO:2000027~regulation of organ morphogenesis	6	4.9E-03	*DVL2, WNT2, PTK7, AHI1, LGR4, TGFB2*	5.4

**Table 2:** The gene ontology of the selective sweep regions’ genes using F_st_ in the Jeju native pigs vs Yorkshire pigs

Term	Count	p-value	Genes	Fold enrichment
GO:1901701~cellular response to oxygen-containing compound	15	3.2E-05	*SASH1, PTPRK, LDLR, PTK7, ESR1, RRAGD, P2RY12, WNT7B, CD86, PTCH1, CIB2, RAPGEF2, KDM6B, GHR, BMP6*	3.8
GO:1901700~response to oxygen-containing compound	17	2.2E-04	*SASH1, PTPRK, LDLR, ESR1, SDK1, PTK7, RRAGD, P2RY12, WNT7B, CD86, GRIN2B, PTCH1, CIB2, RAPGEF2, KDM6B, GHR, BMP6*	2.9
GO:0071396~cellular response to lipid	10	3.1E-04	*SASH1, CD86, WNT7B, LDLR, ESR1, PTK7, PTCH1, PAK1, URI1, BMP6*	4.6
GO:0071310~cellular response to organic substance	23	4.1E-04	*PTPRK, SASH1, LDLR, ESR1, PTK7, DCN, RRAGD, DDIT3, P2RY12, ADAMTS7, MEN1, WNT7B, CD86, IFIT1, CHST11, ROR2, PTCH1, PAK1, CIB2, RAPGEF2, URI1, GHR, BMP6*	2.2
GO:0010033~response to organic substance	26	6.2E-04	*LYPD1, LDLR, PTK7, DCN, RRAGD, MEN1, GRIN2B, CHST11, PAK1, CIB2, RAPGEF2, GHR, SASH1, PTPRK, SDK1, ESR1, DDIT3, ADAMTS7, P2RY12, IFIT1, CD86, WNT7B, ROR2, PTCH1, URI1, BMP6*	2.0
GO:0022008~neurogenesis	18	1.6E-03	*ZNF536, TNIK, FRYL, PTK7, SDK1, PRKG1, KLHL1, P2RY12, GATA2, SLC4A10, WNT7B, PTCH1, RARB, PAK1, RAPGEF2, TCF3, BMP6, IFT140*	2.3
GO:0030182~neuron differentiation	16	1.7E-03	*ZNF536, TNIK, FRYL, PTK7, SDK1, PRKG1, KLHL1, GATA2, SLC4A10, WNT7B, PTCH1, PAK1, RAPGEF2, TCF3, BMP6, IFT140*	2.5
GO:0048699~generation of neurons	17	1.7E-03	*ZNF536, TNIK, FRYL, PTK7, SDK1, PRKG1, KLHL1, P2RY12, GATA2, SLC4A10, WNT7B, PTCH1, PAK1, RAPGEF2, TCF3, BMP6, IFT140*	2.4

**Table 3:** Kyoto encyclopedia of genes and genomes pathway of the selective sweep regions’ genes using F_st_ in the Jeju native pigs vs Berkshire pigs

Term	Count	p-value	Genes	Fold enrichment
ssc04611:Platelet activation	6	0.009	*RASGRP2, GUCY1A3, PIK3CA, PRKG1, MYLK, AKT3*	4.6
ssc04923:Regulation of lipolysis in adipocytes	4	0.019	*PIK3CA, PDE3B, PRKG1, AKT3*	6.9
ssc04150:mTOR signaling pathway	4	0.020	*TSC2, PIK3CA, EIF4E2, AKT3*	6.8
ssc04022:cGMP-PKG signaling pathway	6	0.025	*GUCY1A3, PIK3CA, PDE3B, PRKG1, MYLK, AKT3*	3.6
ssc04917:Prolactin signaling pathway	4	0.028	*ESR1, PIK3CA, CISH, AKT3*	6.0
ssc05205:Proteoglycans in cancer	6	0.040	*WNT2, ESR1, PIK3CA, PTCH1, AKT3, TGFB2*	3.1
ssc04910:Insulin signaling pathway	5	0.041	*TSC2, PIK3CA, PDE3B, EIF4E2, AKT3*	3.8
ssc04390:Hippo signaling pathway	5	0.047	*DVL2, WNT2, MPP5, DLG1, TGFB2*	3.6
ssc02010:ABC transporters	3	0.068	*ABCB11, ABCC1, ABCC8*	6.9
ssc05231:Choline metabolism in cancer	4	0.072	*TSC2, PIP5K1C, PIK3CA, AKT3*	4.1
ssc04660:T cell receptor signaling pathway	4	0.088	*NCK2, PIK3CA, AKT3, DLG1*	3.7
ssc05200:Pathways in cancer	8	0.089	*DVL2, WNT2, RASGRP2, PPARG, PIK3CA, PTCH1, AKT3, TGFB2*	2.0
ssc05217:Basal cell carcinoma	3	0.091	*DVL2, WNT2, PTCH1*	5.8

**Table 4:** Kyoto encyclopedia of genes and genomes pathway of the selective sweep regions’ genes using F_st_ in the Jeju native pigs vs Yorkshire pigs

Term	Count	p-value	Genes	Fold enrichment
ssc05205:Proteoglycans in cancer	6	0.03	*WNT7B, ESR1, PTCH1, DCN, PAK1, ITPR3*	3.5
ssc04080:Neuroactive ligand-receptor interaction	7	0.03	*GABRA2, PTGER3, THRB, RXFP1, GRIN2B, GRIA1, GHR*	2.9
ssc04024:cAMP signaling pathway	6	0.03	*ACOX1, PTGER3, GRIN2B, GRIA1, PTCH1, PAK1*	3.3
ssc05033:Nicotine addiction	3	0.03	*GABRA2, GRIN2B, GRIA1*	10.0
ssc04713:Circadian entrainment	4	0.06	*GRIN2B, GRIA1, PRKG1, ITPR3*	4.5
ssc00510:N-Glycan biosynthesis	3	0.07	*GANAB, ALG6, MGAT5*	6.7
ssc04724:Glutamatergic synapse	4	0.10	*GRIN2B, GRIA1, GLS, ITPR3*	3.6
ssc04730:Long-term depression	3	0.10	*GRIA1, PRKG1, ITPR3*	5.6
