# Supplementary material for: Genome-wide scans for detecting the selection signature of the Jeju-island native pig in Korea
Source: Asian-Australas J Anim Sci. 2019 Jul 1;33(4):539–46. doi: 10.5713/ajas.19.0026 (PMC7054605; doi:10.5713/ajas.19.0026)
Supplement: Supplementary file 1 [file ajas-19-0026-suppl.pdf]

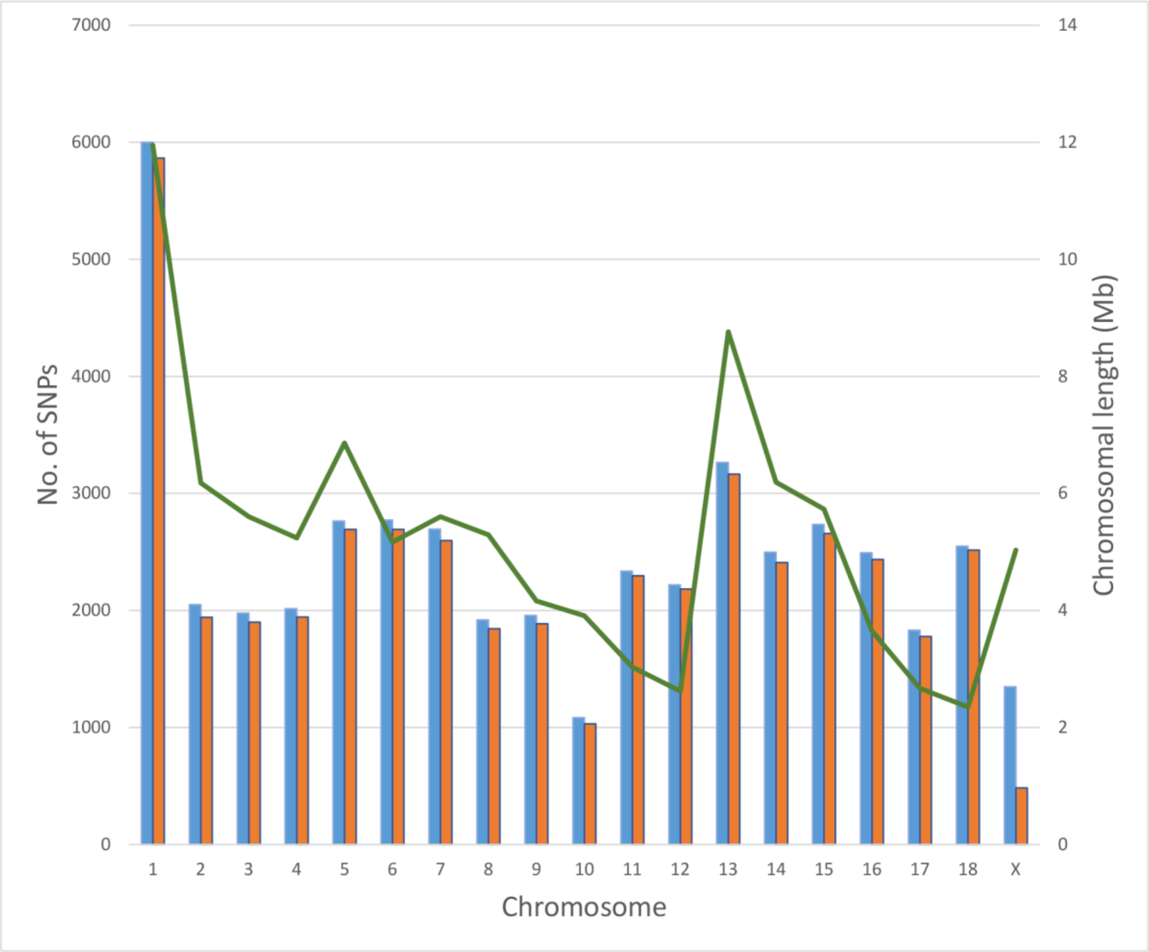

Supplementary Figure S1. The plot of the number of SNPs of merged Jeju native pig-Berkshire (blue bar) and Jeju native pig-Yorkshire (orange bar) SNP data against chromosomes. The green line shows the chromosome length (Mb) across chromosomes. The analysis was conducted after the minor allele frequency (MAF <0.05), Hardy-Weinberg equilibrium (p-value <0.0001) and genotyping call rate (<0.05).

Supplementary File F1. The table of significant gene's SNP list and the allele frequencies in the selective sweep analysis of the Jeju native pig, Berkshire and Yorkshire pigs, which shows the discrepancy of the allele frequency of JNP, Berkshire and Yorkshire pigs.

JNP-Berkshire

CHROM	SNP	POS	Allele1_frequency	Allele1_frequency	Gene name
1	ALGA0001027	13345867	0.95	0.03077	RGS17
1	ALGA0001028	13363514	0.95	0.03077	RGS17
1	ASGA0001083	13385916	0.95	0.03077	RGS17
1	MARC0068793	14312780	0.95	0	ESR1
1	DRGA0000100	14336280	0.05	0.4127	ESR1
1	MARC0018479	14369265	0.89	0	ESR1
1	ASGA0001122	14378568	0.06	0	ESR1
1	ASGA0001125	14398220	0.95	0.2615	ESR1
1	ALGA0001075	14429319	0.89	0.4766	ESR1
1	ASGA0001117	14444305	0.89	0.3516	ESR1
1	ALGA0008126	14471846	0.47	0.03125	ESR1
1	MARC0032404	15923875	0.39	0	PPP1R14C
1	ASGA0001178	16157836	0.94	0	LRP11
1	ASGA0001176	16179177	0.94	0	LRP11
1	ALGA0001463	19798005	0.08	0.2422	FBXO30
1	M1GA0000832	19818935	0.08	0.2812	FBXO30
1	ALGA0001485	19903791	0.75	0.3672	FBXO30
1	ASGA0001497	19992800	0.25	0.1538	FBXO30
1	ALGA0001499	20009618	0.25	0.5	FBXO30
1	H3GA0001152	28574065	0.51	0.007692	AHI1
1	H3GA0001153	28587229	0.77	0.2692	AHI1
1	H3GA0001156	28612780	0.66	0.007692	AHI1
1	MARC0064190	28640879	0.78	0.2692	AHI1
1	ALGA0001818	28713939	0.93	0.007692	AHI1
1	DIAS0000754	28728265	0.51	0.007692	AHI1
1	INRA0001220	28753934	0.51	0.007692	AHI1
1	ASGA0002086	34711558	0.84	0.2385	PTPRK
1	DRGA0000459	34804702	0.42	0	PTPRK
1	ALGA0002350	34859535	0.84	0	PTPRK
1	CASI0009448	34899680	0.56	0.1328	PTPRK
1	ASGA0002111	34926988	0.84	0.3672	PTPRK
1	MARC0024599	34960380	0.84	0.2385	PTPRK
1	ASGA0002114	34974604	0.84	0.2385	PTPRK
1	DRGA0000464	35026068	0.84	0	PTPRK
1	H3GA0001359	35046144	0.55	0.2385	PTPRK
1	ALGA0002382	35135488	0.84	0.2385	PTPRK
1	DRGA0000472	35177589	0.84	0	PTPRK
1	ALGA0005353	116133416	0.41	0.007692	PRTG
1	MARC0088490	116134872	0.94	0	PRTG
1	INRA0003607	116201247	0.56	0.06154	PRTG
1	ALGA0005394	117199378	0.59	0.4375	UNC13C
1	ASGA0004149	117234927	0.77	0.07692	UNC13C
1	ALGA0005381	117359842	0.8	0.4297	UNC13C
1	M1GA0001088	117404197	0.2	0	UNC13C

1	ASGA0004152	117481090	0.77	0.4297	UNC13C
1	DIAS0002366	117504062	0.77	0.4375	UNC13C
1	INRA0003660	117588749	0.77	0.4297	UNC13C
1	MARC0000061	120816036	0.23	0.1692	AP4E1
1	MARC0007969	120894943	0.2	0.2231	AP4E1
1	MARC0114325	145008895	0.61	0	TJP1
1	ASGA0004540	145020686	0.61	0	TJP1
1	ALGA0006451	166493808	0.4	0.007692	CORO2B
1	INRA0004735	166516723	0.4	0.007692	CORO2B
1	M1GA0001375	269216647	0.35	0.2891	LRRC8A
2	MARC0029459	7961326	0.92	0.2769	MACROD1
2	MARC0009476	8003008	0.88	0.05469	MACROD1
2	ALGA0056659	8080491	0.92	0.2538	MACROD1
2	H3GA0029101	9336230	0.94	0	ASRGL1
2	MARC0088006	10337349	0.6	0.3828	PAG6
2	ASGA0046788	18563097	0.94	0.08462	HSD17B12
2	ASGA0046771	18834137	0.94	0.1538	TTC17
2	ASGA0094183	18920105	0.06	0.4615	TTC17
2	ALGA0102823	32873401	0.19	0.04615	LGR4
2	H3GA0029779	32971037	0.29	0.007692	LGR4
2	ASGA0047382	33260438	0.87	0.007692	BBOX1
2	MARC0009248	33273142	0.13	0.4453	BBOX1
2	DRGA0010421	33287874	0.13	0.4194	BBOX1
2	MARC0040445	37816515	0.72	0.007692	NELL1
2	ASGA0047572	38009455	0.84	0	NELL1
2	ALGA0102748	38055678	0.28	0.007692	NELL1
2	ASGA0095530	38064349	0.16	0	NELL1
2	MARC0041439	38198675	0.88	0.4688	NELL1
2	MARC0003765	38220149	0.12	0.007692	NELL1
2	MARC0090810	38405397	0.88	0.007692	NELL1
2	ALGA0058399	38616182	0.28	0.007692	NELL1
2	ALGA0058396	38655801	0.28	0.007692	NELL1
2	ALGA0058402	38702675	0.84	0	NELL1
2	ASGA0047589	38773416	0.84	0	SLC6A5
2	ALGA0058421	39372913	0.28	0.007692	NAV2
2	ALGA0058422	39424934	0.84	0.1953	NAV2
2	ALGA0058427	39507554	0.28	0.1923	NAV2
2	ASGA0047606	39602384	0.28	0.007692	NAV2
2	ALGA0058431	39626717	0.28	0.1923	NAV2
2	MARC0110116	41704537	0.2	0.007692	ABCC8
2	DRGA0010462	42507821	0.88	0.4769	SOX6
2	MARC0037824	42921237	0.11	0.007692	SOX6
2	MARC0033906	43028626	0.89	0.007692	SOX6
2	ALGA0058575	44428078	0.6	0.3615	PDE3B
2	DRGA0010485	44455432	0.89	0.007692	PDE3B
2	ALGA0058578	44475243	0.68	0.007692	PDE3B

2	ALGA0059411	60836899	0.48	0.08462	NWD1
2	ALGA0111692	75002238	0.86	0.01587	PIP5K1C
2	ASGA0083070	75064259	0.86	0.007692	CACTIN
2	ASGA0100222	77827058	0.84	0	SHC2
2	ALGA0124318	77827322	0.84	0	SHC2
3	ALGA0061502	24921076	0.8	0.3923	DNAH3
3	ALGA0061500	24934425	0.8	0.3923	DNAH3
3	ALGA0061496	24954080	0.2	0	DNAH3
3	ALGA0061494	24974538	0.59	0.05385	DNAH3
3	INRA0035764	25029796	0.24	0	DNAH3
3	ASGA0050440	25080131	0.41	0.05385	DNAH3
3	H3GA0031647	25106814	0.41	0.05385	DNAH3
3	ISU10000829	25118285	0.21	0.05385	DNAH3
3	MARC0031825	27250823	0.79	0.3231	XYLT1
3	ALGA0061706	27293298	0.24	0.1308	XYLT1
3	ASGA0050560	27345058	0.22	0.3769	XYLT1
3	ALGA0061701	27414980	0.22	0	XYLT1
3	ASGA0050528	28298982	0.67	0	ABCC6
3	ALGA0061647	28354693	0.67	0	ABCC6
3	DRGA0017432	28465823	0.33	0.2769	ABCC1
3	DRGA0010992	28479512	0.33	0.03077	ABCC1
3	ALGA0061626	29014105	0.67	0.06154	MKL2
3	ALGA0061831	33924241	0.51	0	ABAT
3	ASGA0050635	35006617	0.37	0	RBFOX1
3	CASI0009668	35040703	0.46	0.007692	RBFOX1
3	MARC0040887	35109490	0.33	0	RBFOX1
3	ASGA0050640	35160806	0.81	0.007692	RBFOX1
3	CASI0009238	35233155	0.83	0	RBFOX1
3	ALGA0113843	41211568	0.33	0	RAB11FIP3
3	ALGA0062086	47842912	0.88	0	SULT1C3
3	ALGA0062400	56842973	0.91667	0	CNNM4
3	M1GA0015673	85891795	0.05	0.07692	CCDC88A
4	ALGA0064437	6364431	0.95	0.375	KHDRBS3
4	MARC0077026	6434408	0.95	0.05385	KHDRBS3
4	ALGA0103381	7033158	0.93	0.1154	ZFAT
4	MARC0046612	7107299	0.07	0.1077	ZFAT
4	H3GA0033152	7176696	0.93	0.007692	ZFAT
4	ASGA0053040	9655798	0.86	0	ADCY8
4	ASGA0091982	9778056	0.14	0.1308	ADCY8
4	MARC0087412	9778938	0.14	0	ADCY8
4	ASGA0105686	9783482	0.14	0.1308	ADCY8
4	MARC0071305	9788471	0.14	0	ADCY8
4	ALGA0065273	14961107	0.71	0.01538	MTSS1
4	M1GA0016286	14984702	0.57	0.06154	MTSS1
4	ASGA0083985	15000817	0.41	0.1154	MTSS1
4	ASGA0053421	15048715	0.71	0.05385	MTSS1

4	DRGA0011632	15060605	0.29	0.08594	MTSS1
4	M1GA0016298	15103206	0.86	0.04762	MTSS1
4	H3GA0033798	19339624	0.39	0.1154	ENPP2
4	H3GA0033801	19352812	0.9	0.03846	ENPP2
4	ASGA0053674	19418067	0.39	0.1231	ENPP2
4	DIAS0000091	20544843	0.92	0.03077	SAMD12
4	H3GA0056346	20565887	0.92	0.1308	SAMD12
4	H3GA0033935	24812751	0.76	0.3385	CSMD3
4	H3GA0033927	24868936	0.76	0.03846	CSMD3
4	MARC0013292	24901250	0.24	0.1923	CSMD3
4	ASGA0053829	24928135	0.75	0.3047	CSMD3
4	ASGA0053830	24943528	0.24	0.2077	CSMD3
4	M1GA0026008	24975337	0.75	0.3	CSMD3
4	H3GA0055298	24976658	0.75	0.3438	CSMD3
4	H3GA0056013	25021672	0.75	0.4683	CSMD3
4	ASGA0083170	25035795	0.76	0.4609	CSMD3
4	ASGA0096309	25155524	0.76	0	CSMD3
4	DIAS0001467	25324124	0.7755	0.3	CSMD3
4	ALGA0119814	25386252	0.76	0.3	CSMD3
4	MARC0001152	25502147	0.76	0	CSMD3
4	H3GA0056107	25529494	0.24	0.3	CSMD3
4	ALGA0120891	25532327	0.24	0.3047	CSMD3
4	ASGA0088869	25681693	0.24	0.1154	CSMD3
4	ASGA0102716	25705138	0.76	0.4385	CSMD3
4	ASGA0093576	25708148	0.76	0.4453	CSMD3
4	ALGA0065727	25795011	0.24	0.4194	CSMD3
5	ASGA0090298	3862835	0.4	0.09231	FBLN1
5	ASGA0083287	3868357	0.39	0.3828	FBLN1
5	ASGA0090538	3895272	0.83	0	FBLN1
5	MARC0008185	12757494	0.08	0.4538	BTBD11
5	ALGA0068017	12812184	0.14	0.4286	BTBD11
5	ALGA0068019	12846990	0.76	0.01538	BTBD11
5	ASGA0056017	12881328	0.87	0.01538	BTBD11
5	ASGA0056019	12902274	0.76	0.3923	BTBD11
5	ASGA0056015	12940998	0.07	0.125	BTBD11
5	ASGA0056025	12971106	0.76	0	BTBD11
5	ALGA0068027	12986957	0.11	0.3828	BTBD11
5	ASGA0105236	13041286	0.11	0	BTBD11
5	ASGA0098172	13063614	0.83	0.3438	BTBD11
5	ASGA0096967	13065904	0.94	0.06154	BTBD11
5	ASGA0090392	46607148	0.89	0.3516	STK38L
5	MARC0033333	48840214	0.93	0	BCAT1
5	ALGA0070937	82589660	0.82	0.1154	MYBPC1
5	H3GA0036851	82608481	0.18	0	MYBPC1
5	ALGA0119502	87341419	0.88	0.03077	ELK3
6	ASGA0062657	36457355	0.51	0.1231	LONP2

6	MARC0003105	40476678	0.55	0.08462	ZNF536
6	ASGA0062813	40500953	0.55	0.007692	ZNF536
6	ALGA0077012	40614531	0.92	0.09231	ZNF536
6	M1GA0018527	40636265	0.58	0.1077	ZNF536
6	ASGA0062827	40707564	0.08	0.1	ZNF536
6	H3GA0039834	40752431	0.5	0.1	ZNF536
6	H3GA0039979	44818954	0.54	0.01538	CD22
6	ASGA0063086	46136334	0.58	0.4769	ZNF383
6	MARC0088240	46161643	0.58	0.4769	ZNF383
6	ALGA0077244	46195333	0.58	0.09231	ZNF383
6	MARC0069403	46239548	0.58	0.4769	ZNF383
6	ALGA0077250	46284433	0.42	0.375	ZNF383
6	ALGA0081672	134215187	0.72	0.1615	IFI44L
6	MARC0065165	134425345	0.83	0.1231	IFI44L
6	H3GA0042335	134441868	0.72	0.2344	IFI44L
6	H3GA0042344	134499250	0.83	0.3538	IFI44L
6	ALGA0081694	134530642	0.89	0.1462	IFI44L
6	ASGA0066546	134561404	0.26	0.1615	IFI44L
6	MARC0011591	134601907	0.63	0.4538	IFI44L
6	INRA0047312	134661313	0.89	0.05385	IFI44L
6	SIRI0000626	144268213	0.93	0.3047	RPE65
6	ASGA0068255	152322577	0.85	0.1846	CYP2J34
6	ALGA0083365	152337640	0.85	0.07692	CYP2J34
6	ASGA0068256	152404952	0.85	0	CYP2J34
6	M1GA0020072	152425304	0.85	0.007692	CYP2J34
7	ALGA0122957	35765213	0.77	0.03077	LRFN2
7	ALGA0112215	35765416	0.77	0.03077	LRFN2
7	H3GA0055694	35770550	0.77	0.03846	LRFN2
7	H3GA0054084	35839572	0.07	0.02308	LRFN2
7	H3GA0052608	35905041	0.3	0.04615	LRFN2
7	ASGA0069267	36366214	0.93	0.01538	APOBEC2
7	H3GA0044156	37338998	0.76	0.08462	TRERF1
7	M1GA0020245	37367833	0.31	0.06154	TRERF1
7	ALGA0084813	37382667	0.69	0.03077	TRERF1
7	ALGA0084810	37416839	0.24	0.007692	TRERF1
7	ASGA0069283	37437320	0.24	0.02308	TRERF1
7	MARC0004094	37509826	0.31	0.4308	TRERF1
7	ASGA0069300	37534616	0.93	0.007692	TRERF1
7	ALGA0122296	38140836	0.93	0.2385	PTK7
7	MARC0009352	38184758	0.93	0.4231	PTK7
7	MARC0046272	38329902	0.93	0.01538	SLC22A7
7	ALGA0084907	39799286	0.48	0.1797	SUPT3H
7	ALGA0084910	39814545	0.48	0.1769	SUPT3H
7	MARC0021219	39986267	0.24	0	SUPT3H
7	ALGA0084913	40017923	0.24	0	SUPT3H
7	ALGA0084914	40043981	0.24	0.01538	SUPT3H

7	ASGA0069353	40079840	0.93	0.2769	SUPT3H
7	ALGA0084925	40132137	0.69	0.4	SUPT3H
7	H3GA0044272	51043163	0.24	0.1231	ADAMTSL3
7	INRA0049225	51139397	0.56	0.02308	ADAMTSL3
7	MARC0076820	56091520	0.43	0	FBXO22
7	ALGA0085331	56149951	0.54	0.02308	FBXO22
7	ALGA0085351	56798320	0.43	0	SCAPER
7	INRA0049351	57530002	0.43	0	HMG20A
7	H3GA0044504	80878264	0.34	0.1692	RYR3
7	DIAS0001114	80970451	0.63	0.06154	RYR3
7	ALGA0112336	90453886	0.61	0	GPHN
7	MARC0081952	90470878	0.61	0	GPHN
7	INRA0059933	90490016	0.35	0.007692	GPHN
7	INRA0059935	90490274	0.35	0.007692	GPHN
7	INRA0059937	90491201	0.35	0.007692	GPHN
7	ALGA0086151	90491633	0.35	0.007692	GPHN
7	ALGA0086163	90742366	0.61	0.4219	GPHN
7	ALGA0086180	90971716	0.64	0	MPP5
7	INRA0061523	91612713	0.61	0	RAD51B
7	ASGA0106449	91633645	0.61	0	RAD51B
7	H3GA0044673	92024785	0.61	0	RAD51B
7	ALGA0086215	92063241	0.61	0.2308	RAD51B
7	ASGA0070083	92085105	0.61	0.2308	RAD51B
8	MARC0107047	1715082	0.85	0	NOP14
8	MARC0107047	1715082	0.85	0	MFSD10
8	ALGA0088624	3194852	0.85	0.1615	AFAP1
8	ALGA0121985	3699072	0.89	0	SORCS2
8	MARC0097282	10946289	0.36	0.1231	FBXL5
8	ASGA0072264	10985374	0.88	0.01538	FBXL5
8	ASGA0073063	36441465	0.65	0.1	GABRA2
8	ASGA0085965	37072576	0.35	0	GABRB1
8	ALGA0090278	37608638	0.65	0	CORIN
8	ASGA0073102	38450553	0.61	0	FRYL
9	ALGA0095780	59111820	0.73	0.04615	OPCML
9	ASGA0077604	59140960	0.62	0.04615	OPCML
9	ASGA0077608	59197654	0.89	0.1308	OPCML
9	ASGA0077616	59254197	0.27	0.08462	OPCML
10	ASGA0093702	5832148	0.63	0.4462	USH2A
10	M1GA0024198	5842982	0.63	0	USH2A
10	ASGA0103108	5952932	0.95	0.2619	USH2A
10	ASGA0092077	5991322	0.32	0.4062	USH2A
10	ASGA0100696	5999331	0.37	0.4077	USH2A
10	ASGA0090866	6138538	0.37	0.2077	USH2A
10	M1GA0026358	6303226	0.68	0.4	USH2A
10	ALGA0120578	6537450	0.37	0	USH2A
10	ASGA0095253	8315726	0.87	0.2077	TGFB2

10	MARC0112998	8380770	0.93	0	TGFB2
10	ASGA0078796	9010445	0.94	0.2734	LYPLAL1
10	ALGA0096949	9026572	0.93	0	LYPLAL1
10	MARC0007516	9044109	0.93	0	LYPLAL1
10	ASGA0078820	9800618	0.06	0.01538	RAB3GAP2
10	ALGA0097186	16444813	0.06	0.4769	AKT3
10	ALGA0107372	18359575	0.95	0.2	SMYD3
10	MARC0001634	18396728	0.05	0.5	SMYD3
10	H3GA0050474	18442754	0.05	0.5	SMYD3
10	ALGA0097258	18507823	0.95	0.2	SMYD3
10	H3GA0050481	18538946	0.95	0.1692	SMYD3
10	ALGA0097268	18732284	0.05	0.4923	SMYD3
10	ASGA0079078	18758498	0.95	0.06154	SMYD3
10	DRGA0016900	18789937	0.05	0.4154	SMYD3
10	ASGA0079081	18911413	0.05	0.3906	SMYD3
10	INRA0055347	19464613	0.95	0	SUSD4
10	ALGA0097277	19527294	0.95	0.4538	SUSD4
10	ALGA0097573	26687448	0.24	0.1094	PTCH1
10	DRGA0016947	26825286	0.76	0.007692	FANCC
10	ALGA0097585	26851622	0.24	0.07812	FANCC
10	MARC0039970	26878437	0.24	0.07692	FANCC
10	ASGA0079346	27044596	0.24	0.08594	FANCC
12	H3GA0008998	19921287	0.09	0	AARSD1
12	MARC0015418	19929652	0.09	0	AARSD1
12	ASGA0014373	48407318	0.86	0.007692	TSR1
12	ASGA0014373	48407318	0.86	0.007692	SRR
12	MARC0050111	52588363	0.93	0.03077	DVL2
12	ALGA0018936	53003881	0.93	0.1538	DNAH2
12	ALGA0018936	53003881	0.93	0.1538	KDM6B
12	ALGA0018941	53069445	0.93	0.007692	DNAH2
12	ALGA0018941	53069445	0.93	0.007692	KDM6B
12	MARC0056879	53123161	0.3	0.007692	CHD3
12	MARC0066148	53164999	0.93	0.007692	CHD3
12	INRA0010640	57069591	0.92	0	ARHGAP44
12	ASGA0014697	57093104	0.92	0.1	ARHGAP44
12	ALGA0019121	57129085	0.92	0	ARHGAP44
12	ALGA0108239	57170110	0.92	0	ARHGAP44
12	MARC0001937	57187517	0.92	0.4219	ARHGAP44
12	MARC0008876	58746097	0.92	0	TEKT3
12	ALGA0121347	58775367	0.92	0.2969	TEKT3
12	ASGA0103924	58788483	0.92	0.4297	TEKT3
12	ALGA0124343	58788742	0.92	0.4297	TEKT3
13	ASGA0017629	6116605	0.83	0.1923	KCNH8
13	ALGA0022556	6137527	0.41	0.1769	KCNH8
13	M1GA0005481	6172315	0.42	0.05385	KCNH8
13	ALGA0112712	6265136	0.83	0.09231	KCNH8

13	ALGA0022568	6306405	0.83	0.1308	KCNH8
13	ALGA0022580	6333072	0.42	0.1615	KCNH8
13	H3GA0011596	6360417	0.83	0.01538	KCNH8
13	DIAS0002478	15761999	0.21	0.07692	RBMS3
13	ASGA0018586	15787640	0.77	0.007692	RBMS3
13	ALGA0023517	15959123	0.77	0.06923	RBMS3
13	ALGA0023522	15977749	0.23	0.1077	RBMS3
13	ALGA0023514	16001400	0.23	0.1719	RBMS3
13	ASGA0098453	16081245	0.23	0.1641	RBMS3
13	ASGA0018632	16237949	0.26	0.1797	RBMS3
13	ASGA0018618	16259502	0.95	0.1562	RBMS3
13	ALGA0114568	16347684	0.21	0.2778	RBMS3
13	ASGA0082347	16358804	0.74	0.08462	RBMS3
13	DIAS0000483	16366232	0.74	0.08462	RBMS3
13	ASGA0099287	16375349	0.74	0	RBMS3
13	DIAS0000482	16382153	0.74	0.08462	RBMS3
13	DIAS0000481	16391213	0.74	0.08462	RBMS3
13	ALGA0023561	16438461	0.95	0.01538	RBMS3
13	CASI0006143	66002995	0.38	0.2231	MTMR14
13	H3GA0012785	66381089	0.65	0.4385	IRAK2
13	ASGA0019873	68349470	0.35	0	PPARG
13	MARC0090092	68409038	0.62	0.03077	PPARG
13	H3GA0012816	68480783	0.35	0	TSEN2
13	DRGA0004824	68520632	0.2553	0.007692	TSEN2
13	ALGA0025679	75934281	0.37	0.1406	EPHB1
13	M1GA0005933	75991872	0.4	0.007692	EPHB1
13	ASGA0020070	76023681	0.6	0.05385	EPHB1
13	M1GA0005935	76138962	0.33	0.03077	EPHB1
13	ALGA0026782	100674789	0.87	0.4154	PPM1L
13	MARC0049473	100698952	0.87	0.02308	PPM1L
13	ASGA0020956	100732636	0.87	0.1077	PPM1L
13	H3GA0014315	124804664	0.81	0.08462	ST6GAL1
13	MARC0069132	130706867	0.92	0.2	OPA1
13	INRA0017183	131423303	0.08	0	ATP13A3
13	ASGA0105133	133019096	0.08	0.1846	DLG1
13	ASGA0102506	133098193	0.92	0	MELTF
13	ASGA0022902	135071804	0.05	0.2812	ZNF148
13	ALGA0029117	135083935	0.95	0.06923	ZNF148
13	MARC0062473	136627421	0.71	0.1172	MYLK
13	ASGA0023069	136655786	0.71	0.1538	MYLK
13	ALGA0029294	136802563	0.91	0.01538	MYLK
14	ALGA0031760	40511747	0.48	0	RNF10
14	MARC0030903	41433632	0.48	0	UBE3B
14	H3GA0016877	78589359	0.54	0.4375	LRMDA
14	ASGA0026355	78674472	0.11	0.1231	LRMDA
14	H3GA0055380	78696438	0.11	0.25	LRMDA

14	ALGA0032971	78915180	0.57	0	LRMDA
14	ALGA0032979	78990780	0.57	0.3359	LRMDA
14	M1GA0008025	79010106	0.92	0.007692	LRMDA
14	M1GA0008026	79039700	0.46	0.4766	LRMDA
14	H3GA0017127	95971272	0.08	0.2	PCDH15
14	DRGA0006196	96105052	0.95	0.2923	PCDH15
14	ALGA0033585	96118566	0.87	0.1692	PCDH15
14	ALGA0104592	96190449	0.08	0.1923	PCDH15
14	DIAS0003042	96275403	0.87	0	PCDH15
14	DIAS0003683	96275616	0.87	0	PCDH15
14	ALGA0033587	96322862	0.87	0	PCDH15
14	INRA0020365	96352161	0.87	0	PCDH15
14	ALGA0109664	97617356	0.92	0	PRKG1
14	H3GA0017143	97843765	0.08	0.07692	PRKG1
14	ASGA0026782	97876551	0.88	0.2656	PRKG1
14	MARC0060113	98066715	0.6	0.2077	PRKG1
14	ASGA0102648	98113751	0.6	0.2462	PRKG1
14	ASGA0026812	98223758	0.6	0.2422	PRKG1
14	ASGA0026805	98337406	0.34	0.254	PRKG1
15	MARC0068660	8200415	0.4	0	ARHGAP15
15	ASGA0092787	8303476	0.64	0.4	ARHGAP15
15	ALGA0116973	8340755	0.64	0.4	ARHGAP15
15	MARC0059052	8359798	0.36	0.08462	ARHGAP15
15	ALGA0102858	8361799	0.6	0.3692	ARHGAP15
15	ALGA0104695	8402621	0.64	0.4	ARHGAP15
15	ASGA0090877	8466863	0.36	0.3154	ARHGAP15
15	ALGA0113531	8497891	0.24	0.3984	ARHGAP15
15	ASGA0106245	8500002	0.64	0.4127	ARHGAP15
15	ASGA0102210	8514229	0.64	0.4	ARHGAP15
15	ASGA0100146	8519321	0.24	0	ARHGAP15
15	MARC0058421	8535879	0.24	0	ARHGAP15
15	MARC0025924	8542652	0.24	0	ARHGAP15
15	ALGA0110070	8679743	0.36	0.2937	ARHGAP15
15	ASGA0098319	8800073	0.7	0	ARHGAP15
15	ALGA0102560	8808878	0.7	0	ARHGAP15
15	ALGA0104816	45777816	0.86	0	PRIMPOL
15	ALGA0107986	45800571	0.08	0.01538	PRIMPOL
15	MARC0052953	46687771	0.86	0	SORBS2
15	MARC0040700	46688836	0.86	0	SORBS2
15	ALGA0114446	46893592	0.86	0.2154	SORBS2
15	M1GA0008624	65444642	0.47	0.4769	PKP4
15	ASGA0083057	65551644	0.47	0.05385	PKP4
15	ALGA0035509	65587690	0.47	0.05385	PKP4
15	ALGA0035515	65612144	0.47	0.4127	PKP4
15	MARC0092042	65676538	0.53	0	PKP4
15	ASGA0103262	75138271	0.35	0.1154	CERS6

15	ASGA0095515	75256228	0.66	0.1846	CERS6
15	ASGA0086694	75365266	0.19	0.3308	ABCB11
15	ALGA0110359	75417134	0.81	0.1923	ABCB11
15	DIAS0004205	75428640	0.18	0.1	ABCB11
15	DIAS0003613	75435624	0.18	0.1	ABCB11
15	MARC0073390	75522933	0.18	0.1538	ABCB11
15	MARC0060007	76220720	0.18	0.3984	UBR3
16	DIAS0002191	27841549	0.11	0.1077	ZNF131
16	M1GA0010028	37366668	0.5408	0.3	RAB3C
16	ASGA0032655	37395581	0.11	0.03846	RAB3C
16	MARC0039406	37467931	0.89	0.3047	RAB3C
16	MARC0096194	37507074	0.89	0.3047	RAB3C
16	DIAS0004695	37531069	0.89	0.3047	RAB3C
16	ALGA0040409	37570312	0.92	0.3	RAB3C
16	ALGA0040620	40195443	0.92	0.01538	ZSWIM6
16	ALGA0041064	47873970	0.21	0.05385	BDP1
18	ASGA0097743	28988041	0.26	0.06154	WNT2
18	MARC0090486	28998609	0.94	0.03846	WNT2
JNP-Yorkshire					
CHROM	SNP	POS	Allele1_frequency	Allele1_frequency	Gene name
1	MARC0068793	14312780	0.95	0.1913	ESR1
1	DRGA0000100	14336280	0.05	0.3322	ESR1
1	MARC0018479	14369265	0.89	0	ESR1
1	ASGA0001122	14378568	0.06	0.2152	ESR1
1	ASGA0001125	14398220	0.05	0.4628	ESR1
1	ALGA0001075	14429319	0.11	0.2933	ESR1
1	ASGA0001117	14444305	0.89	0.1722	ESR1
1	ALGA0008126	14471846	0.53	0	ESR1
1	H3GA0000902	17328884	0.2	0.2967	SASH1
1	ASGA0001279	17357389	0.2	0.4223	SASH1
1	H3GA0000905	17369153	0.2	0.4567	SASH1
1	ASGA0001286	17399028	0.2	0.3733	SASH1
1	ASGA0001297	17474143	0.2	0.3133	SASH1
1	ALGA0001286	17491407	0.2	0.3433	SASH1
1	ASGA0001318	17584994	0.62	0.198	SASH1
1	ASGA0001319	17598924	0.2	0	SASH1
1	H3GA0000926	17622619	0.36	0.4628	SASH1
1	ALGA0001311	17637973	0.16	0.4626	SASH1
1	ASGA0002086	34711558	0.84	0.1167	PTPRK
1	DRGA0000459	34804702	0.42	0.06623	PTPRK
1	ALGA0002350	34859535	0.84	0.08333	PTPRK
1	CASI0009448	34899680	0.56	0.4503	PTPRK
1	ASGA0002111	34926988	0.16	0.4735	PTPRK
1	MARC0024599	34960380	0.84	0.4901	PTPRK
1	ASGA0002114	34974604	0.84	0.02649	PTPRK
1	H3GA0001359	35046144	0.55	0.4333	PTPRK

1	ALGA0002382	35135488	0.84	0.08333	PTPRK
1	DRGA0000472	35177589	0.84	0.08278	PTPRK
1	DIAS0002497	35373798	0.84	0.08667	THEMIS
1	ASGA0002255	37184924	0.16	0.04667	NCOA7
1	MARC0033459	53385720	0.87	0.02318	CEP162
1	ALGA0003294	57421664	0.87	0.03333	RRAGD
1	ALGA0005394	117199378	0.41	0.2667	UNC13C
1	ASGA0004149	117234927	0.77	0.2315	UNC13C
1	ALGA0005381	117359842	0.2	0.2651	UNC13C
1	ASGA0004152	117481090	0.23	0.2264	UNC13C
1	DIAS0002366	117504062	0.23	0.2233	UNC13C
1	INRA0003660	117588749	0.23	0.1973	UNC13C
1	ASGA0004416	138050612	0.39	0.01987	LRRC28
1	ASGA0004417	138070556	0.39	0.277	LRRC28
1	ALGA0005829	138128675	0.76	0.006623	LRRC28
1	ALGA0005830	138171977	0.37	0.1913	LRRC28
1	ASGA0004620	149860127	0.82	0	FBXO15
1	INRA0004762	168921751	0.15	0.006623	THSD4
1	INRA0004763	168987602	0.77	0.01325	THSD4
1	ASGA0004931	169007648	0.15	0.01325	THSD4
1	MARC0013229	180360863	0.77	0	ATL1
1	M1GA0001216	182139035	0.77	0.01325	TXNDC16
1	ASGA0005117	182380995	0.77	0.01325	STYX
1	DRGA0001861	222440993	0.73	0	FXN
1	ALGA0008230	253382924	0.91	0.02318	INIP
1	MARC0003821	262063644	0.34	0	TTLL11
2	ASGA0046010	7358925	0.87	0.03642	MEN1
2	MARC0087936	11902479	0.84	0.03642	OSBP
2	ASGA0046788	18563097	0.94	0.06711	HSD17B12
2	ASGA0046771	18834137	0.94	0.2584	TTC17
2	ASGA0094183	18920105	0.06	0.06	TTC17
2	ASGA0083356	27292836	0.34	0.06291	KIAA1549L
2	ALGA0117809	27315287	0.34	0.1879	KIAA1549L
2	ASGA0096712	27357895	0.66	0.1167	KIAA1549L
2	ASGA0047097	27473134	0.26	0.1467	KIAA1549L
2	MARC0086971	27507821	0.26	0.3859	KIAA1549L
2	ALGA0057874	27521990	0.3367	0.4437	KIAA1549L
2	ALGA0057879	27556496	0.74	0.4899	KIAA1549L
2	ASGA0098449	29326082	0.84	0.04305	DNAJC24
2	ASGA0047222	30140466	0.86	0.009934	MPPED2
2	MARC0040445	37816515	0.72	0.1689	NELL1
2	ASGA0047572	38009455	0.16	0.4233	NELL1
2	ALGA0102748	38055678	0.72	0.4233	NELL1
2	ASGA0095530	38064349	0.16	0.4128	NELL1
2	MARC0041439	38198675	0.88	0.3833	NELL1
2	MARC0003765	38220149	0.88	0.4833	NELL1

2	MARC0090810	38405397	0.88	0.3467	NELL1
2	ALGA0058399	38616182	0.72	0.4094	NELL1
2	ALGA0058396	38655801	0.72	0.48	NELL1
2	ALGA0058402	38702675	0.84	0	NELL1
2	ASGA0047589	38773416	0.84	0	SLC6A5
2	H3GA0056653	69850669	0.84	0.04305	LDLR
2	ASGA0083070	75064259	0.86	0.04305	CACTIN
2	ALGA0103072	76863103	0.84	0.04305	TCF3
3	H3GA0030974	2923442	0.33	0.2267	SDK1
3	H3GA0030977	2964267	0.73	0.003311	SDK1
3	ASGA0106209	3313496	0.86	0.3926	SDK1
3	ALGA0061414	23577849	0.49	0.18	OTOA
3	ASGA0050528	28298982	0.67	0	ABCC6
3	ALGA0061647	28354693	0.67	0	ABCC6
3	DRGA0017432	28465823	0.33	0.4633	ABCC1
3	DRGA0010992	28479512	0.67	0.2233	ABCC1
3	ALGA0061626	29014105	0.33	0.202	MKL2
3	INRA0036448	53305837	0.62	0.01987	NPAS2
3	DRGA0011285	53365157	0.62	0.04	NPAS2
3	ALGA0062289	53409601	0.38	0.01	NPAS2
3	ALGA0062400	56842973	0.91667	0.006623	CNNM4
4	DIAS0004710	15174493	0.86	0	RNF139
4	H3GA0033935	24812751	0.76	0.4667	CSMD3
4	H3GA0033927	24868936	0.76	0.06711	CSMD3
4	MARC0013292	24901250	0.24	0.0298	CSMD3
4	ASGA0053829	24928135	0.75	0.447	CSMD3
4	ASGA0053830	24943528	0.76	0.4767	CSMD3
4	M1GA0026008	24975337	0.75	0.447	CSMD3
4	H3GA0055298	24976658	0.75	0.4633	CSMD3
4	H3GA0056013	25021672	0.75	0.06711	CSMD3
4	ASGA0083170	25035795	0.76	0.4633	CSMD3
4	ASGA0096309	25155524	0.76	0.06757	CSMD3
4	ALGA0119814	25386252	0.76	0.4628	CSMD3
4	MARC0001152	25502147	0.76	0	CSMD3
4	H3GA0056107	25529494	0.24	0.46	CSMD3
4	ALGA0120891	25532327	0.24	0.4631	CSMD3
4	ASGA0088869	25681693	0.24	0.46	CSMD3
4	ASGA0102716	25705138	0.76	0.04967	CSMD3
4	ASGA0093576	25708148	0.76	0.04305	CSMD3
4	ALGA0065727	25795011	0.24	0.1107	CSMD3
4	MARC0054687	27939866	0.72	0.2248	SYBU
4	H3GA0034019	27962121	0.72	0	SYBU
4	ASGA0053943	27972588	0.72	0.3667	SYBU
4	ALGA0067173	60510869	0.28	0.1333	HNF4G
4	H3GA0034965	60524117	0.72	0	HNF4G
4	M1GA0017119	60549102	0.28	0.4467	HNF4G

4	M1GA0017151	60577245	0.28	0.4195	HNF4G
4	ALGA0067189	60604972	0.58	0.13	HNF4G
5	ALGA0067515	3528614	0.6	0.2914	WNT7B
5	ASGA0055676	3554668	0.83	0	WNT7B
5	ALGA0067548	3644178	0.44	0.3716	ATXN10
5	ASGA0055704	3670164	0.83	0	ATXN10
5	CASI0005935	3729579	0.55	0.2914	ATXN10
5	ALGA0067557	3750613	0.39	0.1225	ATXN10
5	ALGA0067554	3778163	0.16	0.2914	ATXN10
5	ASGA0090298	3862835	0.4	0.2733	FBLN1
5	ASGA0083287	3868357	0.39	0.1433	FBLN1
5	ASGA0090538	3895272	0.83	0	FBLN1
5	ALGA0110783	4208452	0.16	0.2819	NUP50
5	MARC0008185	12757494	0.92	0.3967	BTBD11
5	ALGA0068017	12812184	0.86	0.27	BTBD11
5	ALGA0068019	12846990	0.76	0.01656	BTBD11
5	ASGA0056017	12881328	0.87	0.01656	BTBD11
5	ASGA0056019	12902274	0.76	0.08108	BTBD11
5	ASGA0056025	12971106	0.76	0.08108	BTBD11
5	ALGA0068027	12986957	0.11	0.08389	BTBD11
5	ASGA0105236	13041286	0.11	0.08108	BTBD11
5	ASGA0098172	13063614	0.83	0.2533	BTBD11
5	ASGA0096967	13065904	0.94	0.09732	BTBD11
5	CASI0004235	22752050	0.11	0	MARS
5	MARC0031933	22785482	0.89	0	DDIT3
5	MARC0031933	22785482	0.89	0	MARS
5	MARC0005330	23116517	0.89	0.003311	CTDSP2
5	ASGA0098365	24875860	0.89	0	SLC16A7
5	ALGA0068910	26426245	0.88	0.003311	FAM19A2
5	ALGA0068921	26499301	0.88	0.003311	FAM19A2
5	ASGA0056722	26522668	0.88	0.003311	FAM19A2
5	ALGA0069000	27130973	0.88	0.003311	MON2
5	ALGA0068996	27155477	0.12	0.3851	MON2
5	ALGA0069008	27183072	0.88	0.1954	MON2
5	MARC0032160	27228257	0.88	0.1821	MON2
5	ALGA0069014	27447736	0.12	0.2919	PPM1H
5	MARC0020531	28348510	0.88	0.4228	SRGAP1
5	ASGA0083178	28521911	0.84	0	SRGAP1
5	ASGA0100164	28531463	0.12	0.1107	SRGAP1
5	ASGA0090184	28561383	0.84	0.2685	SRGAP1
5	ALGA0069150	29029343	0.15	0.4184	RASSF3
5	MARC0028391	29059372	0.85	0	RASSF3
5	ASGA0056898	29081639	0.15	0.3919	RASSF3
5	DIAS0003216	44014547	0.89	0.4533	TMTC1
5	CASI0003561	44065568	0.89	0.05	TMTC1
5	MARC0091931	44215506	0.11	0.1174	TMTC1

5	ASGA0057379	45185760	0.89	0.2067	CCDC91
5	MARC0013995	45316274	0.11	0	CCDC91
5	ASGA0057380	45351416	0.89	0.2785	CCDC91
5	MARC0015917	45480229	0.11	0.1141	CCDC91
5	ASGA0099086	45485705	0.89	0.4	CCDC91
5	ALGA0104080	45493176	0.11	0.1167	CCDC91
5	INRA0040157	48566424	0.93	0.003311	CASC1
5	ASGA0057443	48601957	0.07	0.3733	CASC1
5	MARC0033333	48840214	0.93	0	BCAT1
5	ALGA0070206	58652792	0.79	0.3993	GRIN2B
5	ASGA0057629	58672347	0.79	0	GRIN2B
5	ASGA0057632	58712778	0.21	0.3	GRIN2B
5	H3GA0036502	58758618	0.21	0.2987	GRIN2B
5	MARC0036149	58794358	0.21	0.2953	GRIN2B
5	ASGA0057650	58851471	0.21	0.3624	GRIN2B
5	MARC0036431	58914547	0.21	0.2365	GRIN2B
5	MARC0017185	79798036	0.83	0.104	CHST11
5	ALGA0070877	79810840	0.83	0.01656	CHST11
5	DRGA0012596	79843623	0.17	0.01987	CHST11
5	DRGA0012606	82031954	0.82	0.01656	PARPBP
5	ALGA0070937	82589660	0.82	0.01712	MYBPC1
5	H3GA0036851	82608481	0.18	0	MYBPC1
5	DRGA0012612	82853642	0.82	0.01656	UTP20
5	ALGA0070951	82891929	0.82	0.01656	UTP20
5	MARC0024280	83003977	0.82	0.003311	SLC5A8
5	MARC0109801	83126936	0.82	0.1033	ANO4
5	ALGA0070974	83189686	0.82	0.1033	ANO4
5	ASGA0058207	83278847	0.82	0	ANO4
5	ASGA0058210	83310705	0.82	0.09	ANO4
5	ASGA0058213	83369866	0.82	0.09	ANO4
5	ALGA0070997	83440541	0.82	0.003311	ANO4
5	INRA0040532	83464531	0.82	0.09	ANO4
5	MARC0055409	84197783	0.18	0.2733	ANKS1B
5	ALGA0071031	84239151	0.18	0.2733	ANKS1B
5	ASGA0058239	84277803	0.82	0.01656	ANKS1B
5	ASGA0058240	84471841	0.88	0.003311	ANKS1B
5	ASGA0058243	84662548	0.88	0.2748	ANKS1B
5	ASGA0058242	84704827	0.88	0.01656	ANKS1B
5	ALGA0071041	85011406	0.88	0.003311	ANKS1B
5	MARC0008127	94461626	0.88	0.35	CEP290
6	ASGA0062500	31570456	0.85	0.01325	RPGRIP1L
6	ASGA0062690	37204050	0.46	0	ITFG1
6	MARC0003105	40476678	0.55	0	ZNF536
6	ASGA0062813	40500953	0.55	0	ZNF536
6	ALGA0077012	40614531	0.92	0	ZNF536
6	M1GA0018527	40636265	0.58	0	ZNF536

6	ASGA0062827	40707564	0.08	0	ZNF536
6	H3GA0039834	40752431	0.5	0	ZNF536
6	ALGA0081473	131763135	0.85	0.4433	ADGRL2
6	H3GA0042255	131784048	0.85	0.4396	ADGRL2
6	ALGA0081481	131807843	0.15	0.4333	ADGRL2
6	INRA0047144	131905994	0.85	0.0298	ADGRL2
6	ASGA0066424	132299260	0.85	0.4358	ADGRL2
6	ALGA0081516	132353947	0.85	0.4094	ADGRL2
6	ALGA0081672	134215187	0.28	0.38	IFI44L
6	MARC0065165	134425345	0.83	0.2939	IFI44L
6	H3GA0042335	134441868	0.28	0.1779	IFI44L
6	H3GA0042344	134499250	0.17	0.4831	IFI44L
6	ALGA0081694	134530642	0.89	0.3061	IFI44L
6	ASGA0066546	134561404	0.74	0.4597	IFI44L
6	MARC0011591	134601907	0.37	0.2838	IFI44L
6	INRA0047312	134661313	0.89	0	IFI44L
6	ASGA0067171	141996996	0.33	0.4833	PTGER3
6	ASGA0067905	149095890	0.91	0.003311	ALG6
6	ASGA0068021	149783092	0.81	0	DOCK7
6	INRA0048453	149911431	0.93	0.3867	DOCK7
6	H3GA0043369	149939452	0.08	0.42	DOCK7
7	ASGA0068564	801581	0.25	0	GMDS
7	SIRI0001081	955995	0.57	0	GMDS
7	ALGA0083796	993283	0.25	0	GMDS
7	ALGA0083823	1047733	0.61	0.1	GMDS
7	ALGA0083855	1193806	0.29	0.1933	GMDS
7	DRGA0014803	5072479	0.77	0	BMP6
7	ALGA0083486	5103547	0.23	0.003311	BMP6
7	ALGA0083498	5190799	0.27	0.4793	BMP6
7	ASGA0069039	29817247	0.63	0.04967	IP6K3
7	H3GA0044009	29845111	0.87	0.04967	IP6K3
7	H3GA0044009	29845111	0.87	0.04967	ITPR3
7	MARC0064791	29869965	0.24	0.3154	IP6K3
7	MARC0064791	29869965	0.24	0.3154	ITPR3
7	ALGA0118312	36896149	0.3	0.2785	TFEB
7	ASGA0093910	36899046	0.3	0.2584	TFEB
7	ASGA0100430	36902576	0.85	0.01987	TFEB
7	MARC0083807	36905893	0.07	0.3707	TFEB
7	ALGA0122296	38140836	0.93	0.0298	PTK7
7	MARC0009352	38184758	0.07	0.2333	PTK7
7	MARC0046272	38329902	0.93	0.01656	SLC22A7
7	ALGA0084907	39799286	0.48	0.006623	SUPT3H
7	ALGA0084910	39814545	0.48	0.1309	SUPT3H
7	MARC0021219	39986267	0.24	0	SUPT3H
7	ALGA0084913	40017923	0.24	0	SUPT3H
7	ALGA0084914	40043981	0.24	0	SUPT3H

7	ASGA0069353	40079840	0.07	0.245	SUPT3H
7	ALGA0084925	40132137	0.31	0.1599	SUPT3H
7	MARC0076820	56091520	0.43	0	FBXO22
7	ALGA0085331	56149951	0.46	0.106	FBXO22
7	ALGA0085351	56798320	0.43	0	SCAPER
8	M1GA0020840	1612240	0.74	0	SH3BP2
8	ASGA0073063	36441465	0.35	0.3255	GABRA2
8	ASGA0073102	38450553	0.39	0.2667	FRYL
8	ASGA0073272	48203983	0.06	0	RAPGEF2
8	ALGA0091308	70860525	0.94	0.02667	PARM1
8	ALGA0091294	70878493	0.61	0.3356	PARM1
9	ASGA0075356	11848325	0.38	0.2517	PAK1
9	ALGA0093134	11858708	0.34	0.2185	PAK1
9	ALGA0093152	11941744	0.8	0	PAK1
9	MARC0013253	17686438	0.58	0.2053	DLG2
9	ALGA0093456	17811472	0.84	0.4467	DLG2
9	H3GA0047968	17836349	0.74	0	DLG2
9	INRA0052822	17972781	0.17	0.003311	DLG2
9	ASGA0075556	18245567	0.83	0.4467	DLG2
10	ASGA0093702	5832148	0.37	0.39	USH2A
10	M1GA0024198	5842982	0.63	0.21	USH2A
10	ASGA0092077	5991322	0.68	0.22	USH2A
10	ASGA0100696	5999331	0.63	0.4262	USH2A
10	ASGA0090866	6138538	0.37	0.1733	USH2A
10	M1GA0026358	6303226	0.32	0.1733	USH2A
10	ALGA0120578	6537450	0.37	0.4631	USH2A
10	MARC0013694	12245367	0.25	0.1126	RGS7
10	ASGA0101660	12246767	0.75	0.2767	RGS7
10	ALGA0097182	16239563	0.94	0.15	SDCCAG8
10	H3GA0050438	17477861	0.89	0	HNRNPU
10	ALGA0097306	20978732	0.95	0	NEK7
10	MARC0091906	20991435	0.95	0	NEK7
10	ALGA0097573	26687448	0.24	0.09333	PTCH1
10	ASGA0079354	27175533	0.24	0.4669	C9orf3
10	ALGA0097610	27196942	0.24	0.4633	C9orf3
10	ISU10000740	27237931	0.24	0.4667	C9orf3
10	ALGA0097617	27352273	0.76	0.3456	C9orf3
10	ASGA0079369	27429416	0.76	0.4067	C9orf3
10	ALGA0097620	27442071	0.76	0	C9orf3
10	ASGA0079373	27454936	0.76	0.2635	C9orf3
10	MARC0046857	43251114	0.94	0	CUBN
10	H3GA0050799	43325649	0.1	0.47	CUBN
10	ASGA0079716	43356445	0.94	0.223	CUBN
10	MARC0005447	53314039	0.43	0.05629	MLLT10
10	MARC0041765	53636205	0.43	0.35	NEBL
10	H3GA0055288	53640822	0.57	0.02649	NEBL

10	H3GA0051108	53776647	0.57	0	NEBL
10	ASGA0080257	53821231	0.57	0.1233	NEBL
10	ALGA0098680	53865344	0.57	0.003311	NEBL
11	ALGA0116109	11822418	0.11	0.4631	DCLK1
11	ALGA0106245	11888058	0.89	0.3733	DCLK1
11	H3GA0006023	12110297	0.89	0.006623	DCLK1
11	ALGA0013180	42063092	0.79	0.4801	KLHL1
11	H3GA0006570	42087181	0.06	0.3767	KLHL1
11	ASGA0010012	42134943	0.79	0.1007	KLHL1
11	H3GA0056213	42308503	0.84	0.4189	KLHL1
11	H3GA0052947	42331867	0.84	0.4189	KLHL1
12	ASGA0094509	5282063	0.14	0.1067	RNF157
12	H3GA0056147	5300911	0.17	0.26	RNF157
12	ALGA0017188	5451693	0.83	0	ACOX1
12	ALGA0103433	24365103	0.42	0.46	SKAP1
12	MARC0006151	24387051	0.55	0.4362	SKAP1
12	ALGA0121571	24409351	0.87	0.05369	SKAP1
12	ASGA0101242	24418411	0.45	0.45	SKAP1
12	H3GA0009055	24440989	0.42	0.1333	SKAP1
12	M1GA0004187	24496153	0.09	0.07616	SKAP1
12	ASGA0013894	24602938	0.54	0.4564	SKAP1
12	ASGA0013896	24617723	0.51	0.3459	SKAP1
12	ALGA0018936	53003881	0.93	0.1655	DNAH2
12	ALGA0018936	53003881	0.93	0.1655	KDM6B
12	ALGA0018941	53069445	0.93	0.07718	DNAH2
12	ALGA0018941	53069445	0.93	0.07718	KDM6B
12	MARC0056879	53123161	0.3	0.07718	CHD3
12	MARC0066148	53164999	0.93	0.0777	CHD3
12	CASI0006548	54452578	0.92	0.06376	STX8
12	SIRI0000008	54794036	0.08	0.4033	GAS7
12	DRGA0003883	54820614	0.46	0.4396	GAS7
12	ASGA0014601	54849268	0.92	0.06376	GAS7
13	ASGA0018357	12371181	0.53	0.2674	RARB
13	ASGA0093900	19237526	0.8	0.07667	UBP1
13	ASGA0104019	19262923	0.2	0.07383	UBP1
13	ASGA0102131	19266623	0.45	0.07667	UBP1
13	ALGA0023865	20985135	0.37	0	ARPP21
13	MARC0050565	21697061	0.21	0.03333	LRRFIP2
13	H3GA0012311	21725408	0.5	0.453	LRRFIP2
13	ASGA0018957	21737213	0.5	0.4567	LRRFIP2
13	ALGA0023947	21762434	0.79	0	LRRFIP2
13	ALGA0023950	21778084	0.5	0.4533	LRRFIP2
13	INRA0014565	71524897	0.73	0	EFCC1
13	MARC0054025	71995864	0.73	0	GATA2
13	ASGA0100477	72005343	0.73	0.08725	EEFSEC
13	MARC0052003	72011727	0.73	0	EEFSEC

13	MARC0002683	72028276	0.7	0	EEFSEC
13	INRA0014612	73495852	0.73	0	ACPP
13	ASGA0020010	73521538	0.4	0.3867	ACPP
13	MARC0023014	73565722	0.73	0.3867	ACPP
13	MARC0049594	89448502	0.75	0	CP
13	ALGA0026278	91492141	0.75	0	P2RY12
13	ALGA0026278	91492141	0.75	0	MED12L
13	MARC0114643	91521625	0.75	0	P2RY12
13	MARC0114643	91521625	0.75	0	MED12L
13	ALGA0026645	97929114	0.15	0.1342	RSRC1
13	ASGA0020797	97950816	0.15	0.1544	RSRC1
13	ASGA0020802	97973034	0.85	0.4732	RSRC1
13	INRA0015517	98016950	0.15	0	RSRC1
13	ASGA0020810	98038040	0.15	0.06954	RSRC1
13	ALGA0026672	98059134	0.15	0.06667	RSRC1
13	ASGA0020816	98079027	0.15	0.06954	RSRC1
13	MARC0095572	98209215	0.15	0	RSRC1
13	H3GA0013414	98249700	0.15	0.06954	RSRC1
13	ALGA0026686	98319540	0.15	0.2833	RSRC1
13	ALGA0026782	100674789	0.87	0.198	PPM1L
13	MARC0049473	100698952	0.87	0	PPM1L
13	ASGA0020956	100732636	0.87	0.0604	PPM1L
13	M1GA0006222	109037467	0.8	0.003311	SKIL
13	MARC0027566	109057592	0.2	0.4233	SKIL
13	ASGA0021380	109651091	0.82	0.1014	TNIK
13	ALGA0027315	109697541	0.18	0.3188	TNIK
13	ALGA0027320	109709542	0.82	0.1107	TNIK
13	DIAS0000114	109815184	0.82	0.1122	TNIK
13	H3GA0013765	109845984	0.82	0.1141	TNIK
13	ASGA0021395	109859081	0.82	0.1047	TNIK
13	MARC0011217	109928627	0.79	0	TNIK
13	ASGA0021401	109969089	0.82	0.42	TNIK
13	ASGA0021403	110004343	0.82	0.1081	TNIK
13	ALGA0027351	110023362	0.82	0.1115	TNIK
13	MARC0007903	110606323	0.78	0.009934	FNDC3B
13	ASGA0021414	110832479	0.81	0.3289	FNDC3B
13	ALGA0027556	113535411	0.37	0.04305	NAALADL2
13	ALGA0027558	113546956	0.37	0.02667	NAALADL2
13	MARC0008796	113609363	0.37	0.1074	NAALADL2
13	ASGA0021579	113633442	0.63	0.06667	NAALADL2
13	MARC0001009	113651429	0.63	0.01325	NAALADL2
13	ALGA0027570	113739980	0.63	0.1182	NAALADL2
13	H3GA0013900	113883126	0.37	0.1192	NAALADL2
13	H3GA0013916	114089519	0.37	0.006623	NAALADL2
13	ALGA0027610	114117194	0.37	0.02069	NAALADL2
13	MARC0037393	134203838	0.22	0.4567	MUC20

13	DBWU0000729	134252015	0.73	0.06954	MUC20
13	ALGA0029459	138439538	0.76	0	CD86
14	M1GA0007271	2828502	0.21	0.1745	ROR2
14	M1GA0007274	2876460	0.21	0.2053	ROR2
14	MARC0027217	2975876	0.48	0.3667	ROR2
14	ALGA0030190	6581244	0.73	0	PIWIL2
14	SIRI0000911	6608171	0.73	0	PIWIL2
14	ASGA0025191	30188250	0.72	0.1622	KNTC1
14	ALGA0109285	30230265	0.72	0	KNTC1
14	ALGA0123559	30237457	0.28	0.4561	KNTC1
14	ALGA0031434	30237479	0.28	0.4561	KNTC1
14	H3GA0016329	57539313	0.13	0.3867	PCNX2
14	ALGA0031986	57647715	0.87	0.01667	PCNX2
14	ALGA0031987	57680844	0.87	0.1755	PCNX2
14	DRGA0005871	57747099	0.87	0	PCNX2
14	MARC0065548	60269113	0.87	0.03378	TAF5L
14	ALGA0032035	60296185	0.53	0.4333	TAF5L
14	ALGA0032070	61894232	0.46	0.02	BICC1
14	H3GA0016357	61906403	0.41	0.02	BICC1
14	INRA0019469	61925570	0.41	0.003311	BICC1
14	ALGA0032074	61941547	0.87	0	BICC1
14	MARC0092341	62000754	0.13	0.02	BICC1
14	ALGA0032084	62118993	0.87	0.03	BICC1
14	H3GA0055981	71245453	0.75	0.01342	HERC4
14	MARC0007933	71250247	0.76	0.01333	HERC4
14	ALGA0109664	97617356	0.92	0.3154	PRKG1
14	H3GA0017143	97843765	0.08	0.0596	PRKG1
14	ASGA0026782	97876551	0.88	0.1846	PRKG1
14	MARC0060113	98066715	0.4	0.3113	PRKG1
14	ASGA0102648	98113751	0.6	0.4899	PRKG1
14	ASGA0026812	98223758	0.6	0.19	PRKG1
14	ASGA0026805	98337406	0.34	0.19	PRKG1
14	MARC0048832	101186877	0.86	0.18	LIPA
15	MARC0088620	3005254	0.19	0.1722	LYPD6B
15	H3GA0053053	3110815	0.81	0.03311	LYPD6B
15	M1GA0026382	3126262	0.55	0.3733	LYPD6B
15	MARC0015744	3130922	0.81	0	LYPD6B
15	ASGA0100675	3155479	0.81	0.4567	LYPD6B
15	M1GA0024473	3158965	0.81	0.48	LYPD6B
15	MARC0068660	8200415	0.4	0.1918	ARHGAP15
15	ASGA0092787	8303476	0.36	0	ARHGAP15
15	ALGA0116973	8340755	0.64	0.3893	ARHGAP15
15	MARC0059052	8359798	0.36	0	ARHGAP15
15	ALGA0102858	8361799	0.4	0.2013	ARHGAP15
15	ALGA0104695	8402621	0.36	0.2748	ARHGAP15
15	ASGA0090877	8466863	0.36	0.4833	ARHGAP15

15	ALGA0113531	8497891	0.24	0.4082	ARHGAP15
15	ASGA0106245	8500002	0.64	0.4082	ARHGAP15
15	ASGA0100146	8519321	0.24	0	ARHGAP15
15	MARC0058421	8535879	0.24	0.3061	ARHGAP15
15	MARC0025924	8542652	0.24	0.1905	ARHGAP15
15	ALGA0110070	8679743	0.36	0.2703	ARHGAP15
15	ASGA0098319	8800073	0.7	0.1887	ARHGAP15
15	ALGA0102560	8808878	0.7	0.19	ARHGAP15
15	ASGA0094908	17684737	0.43	0.1067	MGAT5
15	MARC0065967	17800807	0.57	0.4933	MGAT5
15	ALGA0034691	17894448	0.57	0.4633	MGAT5
15	DIAS0000453	17917275	0.57	0.49	MGAT5
15	ALGA0104816	45777816	0.86	0	PRIMPOL
15	ALGA0107986	45800571	0.92	0.3818	PRIMPOL
15	MARC0052953	46687771	0.86	0.08609	SORBS2
15	MARC0040700	46688836	0.86	0	SORBS2
15	ALGA0114446	46893592	0.86	0.08609	SORBS2
15	ASGA0088724	67095178	0.28	0.009934	ITGB6
15	H3GA0053788	67141698	0.28	0.01325	ITGB6
15	MARC0083831	67166532	0.82	0.01325	ITGB6
15	ASGA0084849	68395239	0.81	0.09272	SLC4A10
15	ASGA0099903	68437045	0.82	0.1767	SLC4A10
15	M1GA0024540	68449792	0.81	0.09272	SLC4A10
15	MARC0081365	68530065	0.18	0.01	SLC4A10
15	MARC0052100	68541942	0.28	0.05705	SLC4A10
15	H3GA0052889	68582740	0.18	0.104	SLC4A10
15	ASGA0101526	68583081	0.19	0.1367	SLC4A10
15	ASGA0028666	74446286	0.18	0.4459	STK39
15	MARC0020951	74563663	0.82	0.3467	STK39
15	DBWU0000033	74620551	0.82	0.1159	STK39
15	MARC0081527	74657757	0.81	0.3733	STK39
15	ASGA0104284	74662713	0.18	0.02649	STK39
15	ASGA0103262	75138271	0.35	0.34	CERS6
15	ASGA0095515	75256228	0.66	0.3411	CERS6
15	ASGA0086694	75365266	0.81	0.2133	ABCB11
15	ALGA0110359	75417134	0.19	0.2867	ABCB11
15	DIAS0004205	75428640	0.18	0.01325	ABCB11
15	DIAS0003613	75435624	0.18	0.01333	ABCB11
15	MARC0073390	75522933	0.82	0.4167	ABCB11
15	MARC0096269	94384669	0.85	0.0298	PMS1
15	MARC0012689	94404857	0.51	0.02649	PMS1
15	MARC0107785	94442844	0.34	0	PMS1
15	ALGA0113439	94451345	0.34	0	PMS1
15	MARC0001462	94453726	0.36	0.003311	PMS1
15	ALGA0122144	94464352	0.51	0.1033	PMS1
15	ALGA0036235	95529130	0.83	0.003311	GLS

15	ALGA0036243	95559180	0.83	0.4295	GLS
16	DIAS0001336	27128290	0.7	0.1821	GHR
16	H3GA0020425	27300895	0.81	0.3986	GHR
16	ALGA0039634	27329622	0.89	0.3255	GHR
16	ALGA0039628	27373527	0.89	0	GHR
16	ASGA0031989	27389510	0.11	0.4966	GHR
16	M1GA0010028	37366668	0.4592	0.3596	RAB3C
16	ASGA0032655	37395581	0.11	0.4831	RAB3C
16	MARC0039406	37467931	0.11	0.4831	RAB3C
16	MARC0096194	37507074	0.11	0.4799	RAB3C
16	DIAS0004695	37531069	0.11	0.45	RAB3C
16	ALGA0040409	37570312	0.08	0.4267	RAB3C
16	ALGA0042315	64477735	0.52	0.4	EBF1
16	ASGA0034291	64503112	0.69	0.404	EBF1
16	INRA0026235	64542272	0.69	0	EBF1
16	ASGA0034292	64584851	0.69	0.15	EBF1
16	ASGA0034294	64667698	0.89	0.01987	EBF1
16	DIAS0000883	64721179	0.11	0.2633	EBF1
16	MARC0028986	64757801	0.18	0.02	EBF1
16	MARC0046315	66455084	0.82	0.2185	SGCD
16	H3GA0021974	66604532	0.89	0.01987	SGCD
16	MARC0015528	66664413	0.72	0.01987	SGCD
16	DRGA0007711	66690145	0.89	0.202	SGCD
16	ALGA0042385	68777145	0.7	0	GALNT10
16	DRGA0007722	68821474	0.7083	0	GALNT10
16	DRGA0007723	68854659	0.7	0	GALNT10
16	DRGA0007725	69150213	0.89	0.01987	FAM114A2
16	ASGA0034355	69368366	0.89	0.01987	GRIA1
16	H3GA0021996	69391569	0.89	0.01987	GRIA1
16	ASGA0034357	69434497	0.89	0.02	GRIA1
16	MARC0025572	69481673	0.89	0.01987	GRIA1
16	ALGA0042399	69517503	0.7	0.01987	GRIA1
16	ASGA0034364	69537620	0.89	0.01987	GRIA1
16	INRA0026325	69561175	0.89	0.01987	GRIA1
16	ASGA0034366	69624352	0.72	0.01987	GRIA1
16	H3GA0022004	69640525	0.89	0.01987	GRIA1
16	MARC0033686	69676359	0.72	0.01987	GRIA1
16	MARC0006751	71727030	0.73	0	SLC36A3
17	ALGA0047654	38691276	0.32	0.4866	UQCC1
17	ALGA0047653	38715913	0.17	0	UQCC1
17	ASGA0103358	38771285	0.17	0.07285	UQCC1
